# A novel treatment strategy using indocyanine green for transarterial chemoembolization in BCLC stage C hepatocellular carcinoma

**DOI:** 10.1002/cam4.2671

**Published:** 2019-11-08

**Authors:** Jie Mei, Shao‐Hua Li, Qiao‐Xuan Wang, Xiao‐Ping Zhong, Liang‐He Lu, Anna Kan, Wei Wei, Rong‐Ping Guo

**Affiliations:** ^1^ Department of Hepatobiliary Oncology of the Sun Yat‐sen University Cancer Center Guangzhou P.R. China; ^2^ State Key Laboratory of Oncology in South China Guangzhou P.R. China; ^3^ Collaborative Innovation Center for Cancer Medicine Guangzhou P.R. China; ^4^ Department of Radiation Oncology of the Sun Yat‐sen University Cancer Center Guangzhou P.R. China; ^5^ Department of Burn and Plastic Surgery 2nd Affiliated Hospital of Shantou University Medical College Shantou China

**Keywords:** BCLC stage C, hepatocellular carcinoma, indocyanine green, transarterial chemoembolization, treatment strategy

## Abstract

**Purpose:**

The aim of our study was to propose a strategy based on indocyanine green (ICG) (SBI) to provide better clinical guidelines for transarterial chemoembolization (TACE) treatments for Barcelona clinic liver cancer (BCLC) stage C hepatocellular carcinoma (HCC) patients.

**Materials and Methods:**

From October 2005 to December 2012, 112 BCLC stage C HCC patients initially treated with TACE were investigated, randomly divided into a training cohort (n = 79) and validation cohort (n = 33). In training group, the patients were grouped based on their 15 minutes ICG retention rate (ICG R15), different chemo drugs and dose of lipidol in TACE. Overall survival (OS) and progression‐free survival (PFS) were analyzed in subgroups. Strategy based on ICG was built and verified in validation group.

**Results:**

For those patients with ICG R15 values >10%, the lipiodol ≤10 mL group showed better survival than the lipiodol >10 mL group. For those patients with ICG R15 values ≤10%, the group that received triple‐drug chemotherapy treatments with lipiodol diameter ratio values between 1 and 3 showed better survival than the other group. Patients who conformed with the SBI had better survival times than those who did not conform with the SBI, in both the training cohort (median OS 10.3 vs 5.1 months; *P* < .001; median PFS, 3.3 vs 2.1 months; *P* = .006) and the validation cohort (median OS 8.9 vs 7.1 months; *P* = .087; median PFS, 6.6 vs 2.3 months; *P* < .001).

**Conclusions:**

The SBI is suitable and may provide survival benefits for TACE treatments in BCLC stage C HCC patients.

## Graphical abstract

In this study, we proposed a new indocyanine green (ICG)‐based treatment strategy, which was suitable and might provide survival benefits for transarterial chemoembolization treatments in Barcelona clinic liver cancer stage C hepatocellular carcinoma patients. For those patients with ICG retention rate (ICG R15) values greater than 10%, the lipiodol dose should be reduced to no more than 10 mL, and for those patients with ICG R15 values less than 10%, maintain the LDR value between 1 and 3 and use a triple chemotherapy drug regimen.

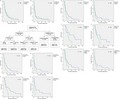

## 1 INTRODUCTION

Hepatocellular carcinoma (HCC) is the most common primary malignancy of the liver, the sixth most common cancer, and the third leading cause of cancer‐related deaths worldwide.^1^ Due to its insidious onset and high malignancy, HCC is often diagnosed at an intermediate or advanced stage and without a chance for radical resection.

Transarterial chemoembolization (TACE) is the most widely used primary therapy for unresectable HCC.^2^ For patients classified as stage C of the Barcelona clinic liver cancer (BCLC) criteria, European and American guidelines recommend that the first line of treatment should be systemic but in everyday clinical practice, especially in Asia; due to the poor efficacy of systemic chemotherapy and the high costs of targeted drugs, TACE remains the most common form of treatment and has demonstrated to effectively improve the prognosis of HCC patients.^2^, ^3^ Moreover, our previous investigations have also shown that TACE can be beneficial for improving the survival of HCC patients with portal vein tumor thrombus.^4^, ^5^ However, because these patients usually have large tumor burdens, poor liver function, and unsatisfied bad basic states, they experience a higher incidence of liver failure after TACE treatments as compared to those of early and intermediate liver cancer patients. Also, as the use of embolic and chemotherapeutic agents varies considerably among medical centers, this often results in inconsistent and unsatisfactory survival outcomes after the TACE treatments. Therefore, a standardized recommendation that can guide TACE treatment strategies in BCLC stage C HCC patients is necessary to improve their prognoses.

Indocyanine green (ICG), a tricarbocyanine dye that binds to albumin and alpha‐1 lipoproteins, is produced in hepatocytes and secreted into the bile. Measuring of the ICG clearance can be used as a quantitative liver function test to represent both parenchymal function and hepatic blood flow.^6^ Early in 1999, Makuuchi et al first suggested that choosing an appropriate surgical hepatectomy should be based on the 15 minutes retention rate of ICG (ICG R15).^7^ In recent years, the ICG R15 has been commonly used and is recognized as an important indicator for evaluating liver reserve function prior to hepatectomies.^8^ However, there have been a few studies on the application of ICG R15 prior to TACE treatment. Shalimar et al have proposed that ICG could play an important role for the prediction of liver failure after TACE treatment.^9^

We designed this current study to comprehensively assess the tumor status and liver function of BCLC stage C HCC patients before TACE treatment and to formulate a treatment strategy for them, with the aim to improve their therapeutic efficacy, reduce the incidence of adverse events, improve their quality of life, and prolong their survival.

## 2 MATERIALS AND METHODS

This study was conducted according to the ethical guidelines of the 1975 Declaration of Helsinki. The analyses of the patients' data have been reviewed and approved by the Institutional Review Board of Sun Yat‐sen University Cancer Center (SYSUCC) (no. B2018‐134‐01).

### 2.1 Patients

In this retrospective study, patients who were diagnosed with HCC from October 2005 to December 2012 at the SYSUCC and had TACE treatments as standard therapy were screened for eligibility. Only patients who satisfied all of the following criteria were enrolled in this study: (a) BCLC stage C HCC, based on the American Association for the Study of Liver Diseases guidelines,^2^, ^10^ diagnosed before the TACE treatment; (b) the patient had not received any anti‐tumor therapies prior to the TACE treatment; (c) absence of other malignant diseases; (d) no brain and/or bone metastases; (e) the patient had at least one contrast‐enhanced imaging examination, such as computed tomography (CT), magnetic resonance imaging (MRI) or ultrasonic contrast (UC), performed within 7 days prior to treatment; and (f) the patient had a complete record of systemic vital organ function tests, including electrocardiogram, liver function, renal function, coagulation function, and ICG examination, performed within 3 days prior to treatment.

A total of 112 patients ultimately satisfied the inclusion criteria and were recruited for the study. Of these, 70% of the cases were randomly selected to be the training cohort (79 cases), and the remaining cases were used as the validation cohort (33 cases). In training group, the patients were divided into the ICG R15 ≤ 10% group (n = 56), which were further divided into a standard (triple‐drug chemotherapy with lipiodol diameter ratio (LDR) values between 1 and 3, n = 40) and a nonstandard subgroup (n = 16), and ICG R15 > 10% group (n = 23), which were further categorized into a lipiodol ≤ 10 mL subgroup (n = 8) and a lipiodol > 10 mL subgroup (n = 15).

### 2.2 ICG clearance examination

A total of 25 mg ICG (Dandong Yichuang Pharmaceutical), dissolved in 5 mL of saline, were injected into a peripheral vein over a span of 10‐20 seconds, at a dose of 0.5 mg/kg. An optical sensor was then placed onto the two sides of the ala of one nostril, and the patients ICG R15 was automatically measured using a pulse spectrophotometry (DDG‐3300K; Nihon Kohden), enabling the continuous measurement of ICG plasma concentrations at the optical peak absorptions for wavelengths 805 and 890 nm at every pulse interval. ICG, being a deep blue‐green dye that binds to serum proteins, is selectively absorbed by the liver and released into the bile in a free form. It is nontoxic, does not participate in the intestine‐liver circulation, and is not excreted by the kidneys. Because of good light absorption, the ICG concentration in the blood can be accurately determined.^11^ All patients were kept hemodynamically stable with percutaneous oxygen levels over 90% during the procedure. Participating treating physicians or operational personnel all received adequate training on to use the device prior to the treatment.

### 2.3 TACE procedure

Angiographies of celiac, hepatic, superior mesenteric, left gastric, and inferior phrenic arteries were performed to identify all of the feeding arteries of the tumor. The chemotherapeutic drug and lipiodol (Lipiodol UltraFluide; Guerbet Laboratories) were mixed into a suspension and injected through the segmental or subsegmental target artery. The chemotherapeutic drugs used generally comprised of at least one of the following four types: platinum (25‐50 mg lobaplatin [Hainan Changan International Pharmaceutical Co. Ltd.], 100‐300 mg carboplatin [Bristol‐Myers Squibb], 50‐150 mg oxaliplatin [Sanof Synthelabo France]), anthracycline (30‐60 mg pirarubicin [Wan Le Pharmaceutical; Shen Zhen Co. Ltd.]), antibiotics (30‐60 mg epirubicin [Pfizer], 4‐10 mg mitomycin C [Zhejiang Hisun Pharmaceutical Co. Ltd.]), or fluorouracil (30‐60 mg Floxuridine [Nantong Jinghua Pharmaceutical Co. Ltd.], 100‐500 mg 5‐fluorouracil [Shanghai Xudong Haipu Pharmaceutical Co. Ltd.]). Similar drugs were not repetitively applied. Absorbable gelatin sponge (AGS) (H32024096; Gelfoam; Hanzhou alc Ltd) 350‐560 µm in diameter or polyvinyl alcohol (PVA) (Cook) 300 µm in diameter was injected in place of lipiodol if necessary. The mixture was infused at a rate of 0.5‐1 mL/min until flow stasis was achieved in the tumor vasculature.

### 2.4 Post‐TACE treatment care and follow‐up

Posttreatment care was conduct routinely in all patients. The end of follow‐up was 31 July 2018. Each follow‐up session consisted of complete physical examination, laboratory tests, such as hematic convention analysis, coagulation function test, serum alpha‐fetoprotein (AFP) and liver function assessment, and an abdominal contrast‐enhanced three‐phase dynamic spiral CT or MRI. Tumor response was defined as complete response, partial response, stable disease, or progression disease, according to the modified response evaluation criteria in solid tumors (mRECIST).^12^ The follow‐up sessions for all patients were performed 30‐60 days after TACE treatment, then once every 3 months for the first 2 years and once every 6 months beyond 2 years. Proper subsequent treatments were performed routinely.

### 2.5 Statistical analysis

Categorical variables were compared using Pearson's *χ* ^2^ test or Fisher's exact test. Variable distributions were described using the mean ± SE, for normally distributed values, and medium and range for nonnormally distributed values. Continuous variables were compared using the Student's *t* test for normally distributed values or the Mann‐Whitney test for skewed distributed values. The survival analysis was calculated using the Kaplan‐Meier method, and differences in the survival curves were analyzed with a log‐rank test. A two‐tailed *P* < .05 was considered statistically significant. All data analyses were performed using the SPSS software, version 25.0 (SPSS Inc).

## 3 RESULTS

### 3.1 Overall clinical characteristics and survival analysis

**Figure 1:**
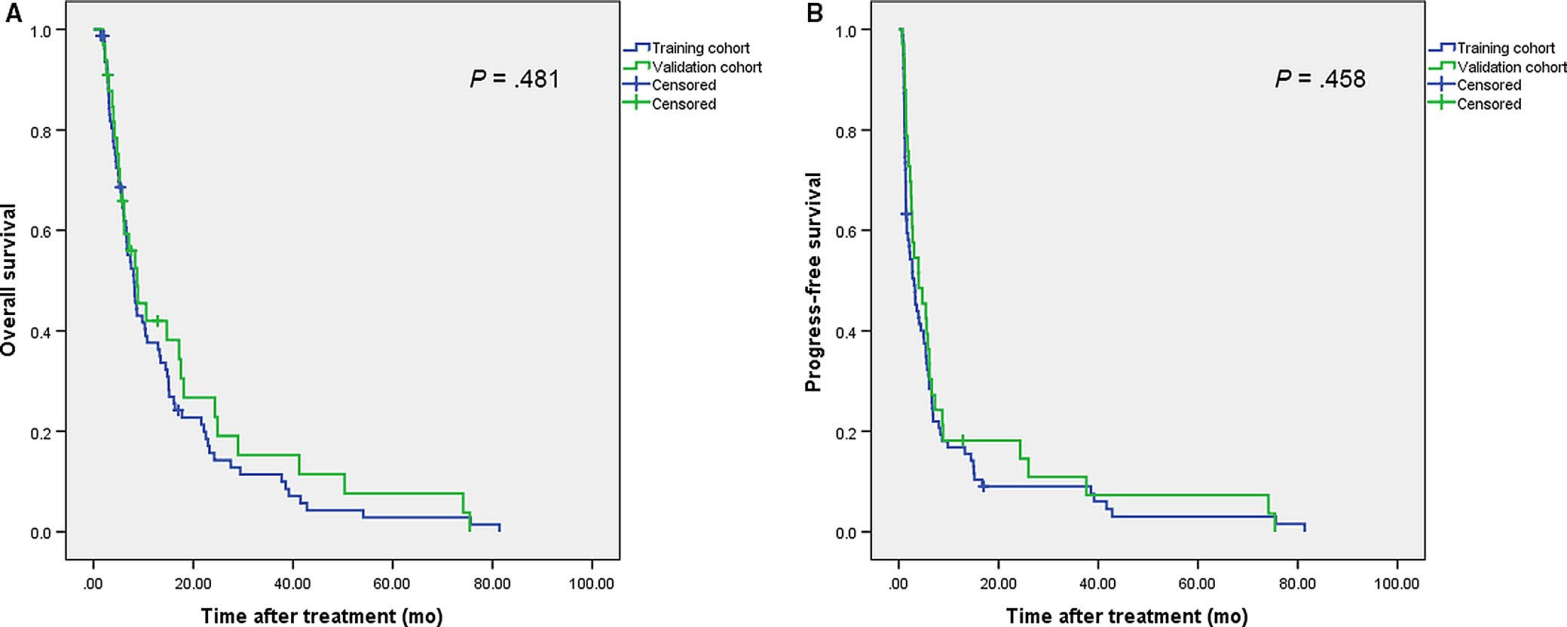
Kaplan‐Meier survival curves for patients in the training and validation cohorts. A, overall survival; (B) progression‐free survival

Between October 2005 and December 2012, 112 patients with BCLC stage C HCC who were initially treated with TACE were randomly divided into a training cohort (n = 79) and a validation cohort (n = 33). The baseline characteristics of all patients are described in Table 1. The clinical characteristics, liver function, tumor characteristics, and treatment modalities between the two cohorts were relatively homogeneous. They had similar tumor therapy responses (*P* = .230), OS (*P* = .481) and progression‐free survival (PFS) (*P* = .458) (Figure 1).The only difference between them was that patients in the validation cohort had a higher average age (mean ± SE 52.3 ± 2.1 years) than the training cohort (mean ± SE 46.6 ± 1.3 years).

**Table 1:** Baseline clinical characteristics and treatment analyses of patients in the training and validation cohorts

Characteristic	Training cohort (n = 79)	Validation cohort (n = 33)	Overall (n = 112)	*P* value
Age (y)^a^	46.6 ± 1.3	52.3 ± 2.1	48.3 ± 1.1	.019
Gender^b^				1.000
Female	8 (10.1%)	29 (87.9%)	100 (89.3%)	
Male	71 (89.9%)	4 (12.1%)	12 (10.7%)	
Maximal diameter of tumor (cm)^a^	10.0 ± 0.4	9.0 ± 0.5	9.7 ± 0.3	.147
Number of tumor (s)^b^				.303
Single	34 (43.0%)	18 (54.5%)	52 (46.4%)	
Multiple	45 (57.0%)	15 (45.5%)	60 (53.6%)	
Tumor distribution^b^				.094
Unilobar	40 (50.6%)	23 (69.7%)	63 (56.3%)	
Bilobar	39 (49.4%)	10 (30.3%)	49 (43.8%)	
Macrovascular invasion^b^				.551
Absent	11 (13.9%)	3 (9.1%)	14 (12.5%)	
Present	68 (86.1%)	30 (90.9%)	98 (87.5%)	
Extrahepatic metastasis^b^				.622
Absent	60 (75.9%)	27 (81.8%)	87 (77.7%)	
Present	19 (24.1%)	6 (18.2%)	25 (22.3%)	
Ascites^b^				.103
Absent or mild	72 (91.1%)	33 (100%)	105 (93.8%)	
Moderate or severe	7 (8.9%)	0 (0%)	7 (6.3%)	
HBsAg^b^				1.000
Negative	8 (10.1%)	4 (12.1%)	12 (10.7%)	
Positive	71 (89.9%)	29 (87.9%)	100 (89.3%)	
PLT (×10^9^/L)^a^	201.6 ± 8.9	198.7 ± 11.1	200.8 ± 7.0	.849
ALT (U/L)^c^	44.2 (10.4‐198.0)	47.9 (14.8‐174.0)	44.9 (10.4‐198.0)	.437
AST (U/L)^c^	71.8 (19.2‐180.0)	56.4 (16.9‐394.5)	64.9 (16.9‐394.5)	.093
ALP (U/L)^c^	135.8 (62.0‐1682.5)	118.0 (55.7‐404.3)	127.9 (55.7‐1682.5)	.430
GGT (U/L)^c^	179.0 (34.0‐179.0)	179.0 (35.3‐485.2)	179.0 (34.0‐794.5)	.381
TBIL (µmol/L)^c^	16.3 (5.0‐50.1)	15.2 (5.9‐70.5)	16.1 (5.0‐70.5)	.379
CRE (µmol/L)^a^	71.4 ± 1.7	71.0 ± 2.1	71.3 ± 1.3	.886
PT (s)^c^	12.5 (10.8‐16.4)	12.4 (10.7‐18.6)	12.5 (10.7‐18.6)	.642
AFP (ng/mL)^c^	6367.0 (1.8‐121 000)	882.4 (2.23‐129 470)	3516.5 (1.86‐129 470)	.224
Child‐Pugh score^b^				.424
5	63 (79.7%)	25 (75.8%)	88 (78.6%)	
6	10 (12.7%)	7 (21.2%)	17 (15.2%)	
≥7	6 (7.6%)	1 (3.0%)	7 (6.3%)	
ICG R15 (%)^c^	5.5 (0.3‐40.1)	7.5 (0.8‐35.0)	6.25 (0.3‐40.1)	.110
Type of chemotherapy drug^b^				.562
1	10 (12.7%)	4 (12.1%)	14 (12.5%)	
2	6 (7.6%)	1 (3.0%)	7 (6.3%)	
3	63 (79.7%)	27 (81.8%)	90 (80.4%)	
4	0 (0%)	1 (3.0%)	1 (0.9%)	
Dose of lipiodol (mL)^c^	15.0 (0‐65.0)	15.0 (5.0‐65.0)	15.0 (0‐65.0)	.605
Usage of PVA/AGS^b^				1.000
No	69 (87.3%)	29 (87.9%)	98 (87.5%)	
Yes	10 (12.7%)	4 (12.1%)	14 (12.5%)	
Tumor response^b^	65 (100%)	30 (100%)	95 (100%)	.230
CR	0 (0%)	0 (0%)	0 (0%)	
PR	6 (9.2%)	4 (13.3%)	10 (10.5%)	
SD	27 (41.5%)	17 (56.7%)	44 (46.3%)	
PD	32 (49.2%)	9 (30.0%)	41 (43.2%)	
NA	14	3	17	
OS (mo)^d^	8.1 (6.2‐8.1)	8.7 (4.4‐13.1)	8.2 (6.78‐9.69)	.481
PFS (mo)^d^	3.1 (1.70‐4.5)	4.0 (1.0‐6.9)	3.2 (2.21‐4.26)	.458

Abbreviations: AFP, alpha‐fetoprotein; AGS, absorbable gelatin sponge; ALP, alkaline phosphatase; ALT, alanine aminotransferase; AST, aspartate aminotransferase; CR, complete response; CRE, creatinine; GGT, glutamyl transpeptidase; HBsAg, hepatitis B surface antigen; ICG R15, retention rate of indocyanine green for 15 minutes; NA, not assessable; OS, overall survival; PD, progression disease; PFS, progress‐free survival; PLT, blood platelet; PR, partial response; PT, prothrombin time; PVA, polyvinyl alcohol; SD, stable disease; TBIL, total bilirubin.

a Mean ± SE

b No. (%).

c Median (range).

d Medium (95% CI).

### 3.2 Clinical characteristics and survival analysis based on ICG R15 in the training cohort

**Figure 2:**
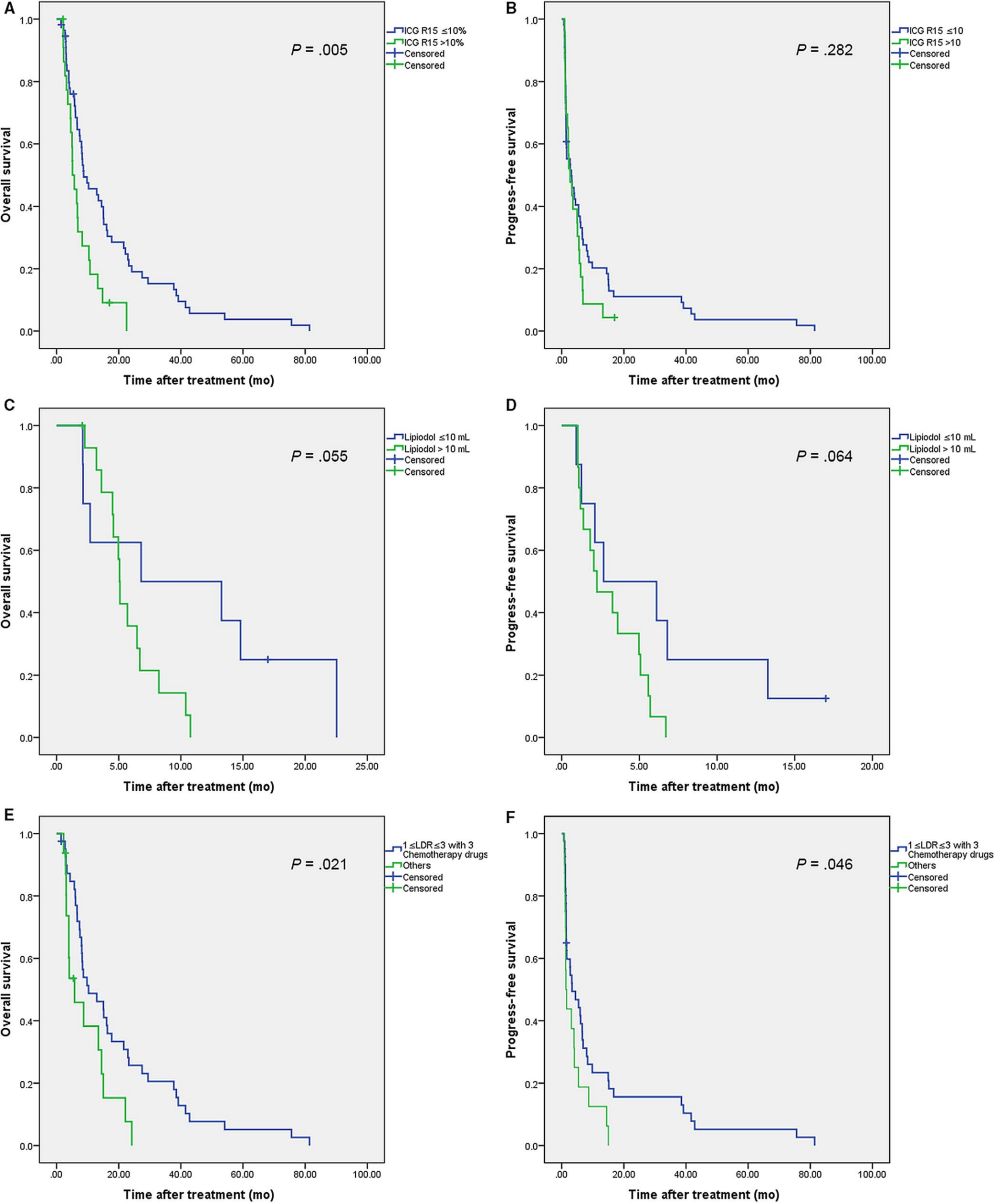
Kaplan‐Meier survival curves for patients with transarterial chemoembolization treatment. A and B, overall survival (OS) (A) and progression‐free survival (PFS) (B) in the indocyanine green (ICG) R15 ≤ 10% and ICG R15 > 10% groups. C,D, OS (C) and PFS (D) in the lipiodol ≤ 10 mL and lipiodol > 10 mL groups. E and F, OS (E) and PFS (F) in the patients with LDR values between 1 to 3, combined with 3 types of chemotherapy drugs, and those with exception

The patients were divided into the ICG R15 ≤ 10% group (n = 56) and ICG R15 > 10% group (n = 23), in view of 10% is recognized as a normal standard value of ICG R15 in clinical application.^13^ The clinical characteristics and treatment analyses of these patients are shown in Table 2. The blood platelet levels was higher, while the aspartate aminotransferase (AST), alkaline phosphatase, glutamyl transpeptidase, and total bilirubin (TBIL) levels were significantly lower in the ICG R15 ≤ 10% group compared with those of the ICG R15 > 10% group. In addition, a higher percentage of patients with moderate‐to‐severe ascites (21.7% vs 3.6%; *P* = .02) and Child‐Pugh scores ≥ 7 (21.7% vs 1.8%; *P* = .017) were observed in the ICG R15 > 10% group as compared to those in the ICG R15 ≤ 10% group. These results indicated that the ICG R15 value can largely represent the liver functions. However, factors related to tumors, such as AFP, maximum tumor diameter, number of lesions, lobular distribution and metastatic status, the use of chemotherapy, and the dose of lipiodol, PVA or AGS, showed no significant differences between these two ICG R15 groups. Survival analyses showed that the median OS for the ICG R15 ≤ 10% and > 10% group was 8.7 and 5.1 months (95% confidence interval [CI]: 3.1‐14.3 vs 3.4‐6.8 months; *P* = .005), respectively. Further, median PFS for the ICG R15 ≤ 10% and >10% group was 3.1 and 2.7 months (95% CI: 0.3‐5.9 vs 0.9‐4.5 months; *P* = .282), respectively. The survival illustrations are shown in Figure 2A,B.

**Table 2:** Characteristics and treatment analyses of patients in the training cohort, based on ICG R15

Characteristic	ICG R15 ≤ 10% (n = 56)	ICG R15 > 10% (n = 23)	*P* value
Age (y)^a^	44.5 ± 1.5	51.8 ± 2.2	.007
Gender^b^			.685
Female	5 (8.9%)	3 (13.0%)	
Male	51 (91.1%)	20 (87.0%)	
Maximal diameter of tumor (cm)^a^	9.8 ± 0.3	10.1 ± 0.9	.708
Number of tumor (s)^b^			.803
Single	25 (44.6%)	9 (39.1%)	
Multiple	31 (55.4%)	14 (60.9%)	
Tumor distribution^b^			.622
Unilobar	27 (48.2%)	13 (56.5%)	
Bilobar	29 (51.8%)	10 (43.5%)	
Macrovascular invasion^b^			1.000
Absent	8 (14.3%)	3 (13.0%)	
Present	48 (85.7%)	20 (87.0%)	
Extrahepatic metastasis^b^			1.000
Absent	43 (76.8%)	17 (73.9%)	
Present	13 (23.2%)	6 (26.1%)	
Ascites^b^			.020
Absent or mild	54 (96.4%)	18 (78.3%)	
Moderate or severe	2 (3.6%)	5 (21.7%)	
HBsAg^b^			.426
Negative	7 (12.5%)	1 (4.3%)	
Positive	49 (87.5%)	22 (95.7%)	
PLT (×10^9^/L)^a^	214.3 ± 10.2	170.9 ± 16.4	.026
ALT (U/L)^c^	44.9 (10.4‐169.2)	43.5 (22.6‐198.0)	.490
AST (U/L)^a^, ^c^	62.25 (19.2‐179.7)	102.0 ± 8.0	.002
ALP (U/L)^c^	119.0 (62.0‐1682.5)	153.4 (64.5‐386.2)	.006
GGT (U/L)^c^	172.5 (34.0‐560.8)	256.2 (79‐794.5)	.027
TBIL (µmol/L)^c^	15.7 (5.0‐50.1)	19.7 (9.8‐48.4)	.010
CRE (µmol/L)^a^	72.9 ± 2.0	67.9 ± 3.2	.184
PT (s)^c^	12.5 (10.8‐16.4）	12.6 (11.5‐15.6)	.821
AFP (ng/mL)^a^	37 759.8 ± 6,476.2	28 774.9 ± 9478.4	.449
Child‐Pugh score^b^			.017
5	47 (83.9%)	16 (69.6%)	
6	8 (14.3%)	2 (8.7%)	
≥7	1 (1.8%)	5 (21.7%)	
Type of chemotherapy drug^b^			.205
1	8 (14.3%)	2 (8.7%)	
2	2 (3.6%)	4 (17.4%)	
3	46 (82.1%)	17 (73.9%)	
Dose of lipiodol (mL)^c^	15.0 (0‐35.0)	15.0 (0‐25.0)	.192
Usage of PVA/AGS^b^			.177
No	47 (83.9%)	22 (95.7%)	
Yes	9 (16.1%)	1 (4.3%)	
Tumor response^b^	48 (100%)	17 (100%)	.489
CR	0 (0%)	0 (0%)	
PR	3 (6.3%)	3 (17.6%)	
SD	21 (43.8%)	6 (35.3%)	
PD	24 (50.0%)	8 (47.1%)	
OS (mo)^d^	8.7 (3.1‐14.3)	5.1 (3.4‐6.8)	.005
PFS (mo)^d^	3.1 (0.3‐5.9)	2.7 (0.9‐4.5)	.282

Abbreviations: AFP, alpha‐fetoprotein; AGS, absorbable gelatin sponge; ALP, alkaline phosphatase; ALT, alanine aminotransferase; AST, aspartate aminotransferase; CRE, creatinine; GGT, glutamyl transpeptidase; HBsAg, hepatitis B surface antigen; ICG R15, retention rate of indocyanine green for 15 minutes; NA, not assessable; OS, overall survival; PD, progression disease; PFS, progress‐free survival; PLT, blood platelet; PR, partial response; PT, prothrombin time; PVA, polyvinyl alcohol; SD, stable disease; TBIL, total bilirubin.

a Mean ± SE.

b No. (%).

c Median (range).

d Medium (95% CI).

### 3.3 Treatment strategy in patients with ICG R15 > 10%

The patients with ICG R15 > 10% were further categorized into a lipiodol ≤ 10 mL subgroup (n = 8) and a lipiodol > 10 mL subgroup (n = 15). The clinical characteristics and treatment analyses of these two groups are shown in Table 3. The primary differences between the two groups were that the patients from the lipiodol > 10 mL subgroup had larger tumor sizes (*P* = .001), lower pre‐interventional levels of TBIL (*P* = .017), and lower prothrombin time (*P* = .024), and higher postinterventional levels of ALT (*P* = .039) and AST (*P* = .039) than patients from the lipiodol ≤ 10 mL subgroup (Figure 2C,D). Patients from the subgroup lipiodol ≤ 10 mL had better OS (median 6.8 months vs 5.1 months; 95% CI: 0‐21.4 months vs 4.8 −5.3 months) and PFS (median 2.7 months vs 2.3 months; 95% CI: 0‐8.2 months vs 0.5‐4.1 months) as compared with those from the subgroup lipiodol > 10 mL; however, these differences were not statistically significant (*P* = .055 and .064, respectively).

**Table 3:** Clinical characteristics and treatment analyses of ICG R15 > 10% patients, grouped by lipiodol dose

Characteristic	Lipiodol ≤ 10 mL (n = 8)	Lipiodol > 10 mL (n = 15)	*P* value
Age (y)^a^	52.8 ± 3.6	51.3 ± 2.9	.767
Gender^b^			1.000
Female	7 (87.5%)	13 (86.7%)	
Male	1 (12.5%)	2 (13.3%)	
Maximal diameter of tumor (cm)^a^	6.3 ± 1.0	12.2 ± 1.0	.001
Number of tumor (s)^b^			.400
Single	2 (25.0%)	7 (46.7%)	
Multiple	6 (75.0%)	8 (53.3%)	
Tumor distribution^b^			.685
Unilobar	4 (50.0%)	9 (60.0%)	
Bilobar	4 (50.0%)	6 (40.0%)	
Macrovascular invasion^b^			.289
Absent	0 (0.0%)	3 (20.0%)	
Present	8 (100.0%)	12 (80.0%)	
Extrahepatic metastasis^b^			.369
Absent	7 (87.5%)	10 (66.7%)	
Present	1 (12.5%)	5 (33.3%)	
Ascites^b^			.297
Absent or mild	5 (62.5%)	13 (86.7%)	
Moderate or severe	3 (37.5%)	2 (13.3%)	
HBsAg^b^			.426
Negative	7 (12.5%)	1 (4.3%)	
Positive	49 (87.5%)	22 (95.7%)	
PLT (×10^9^/L)^a^	152.8 ± 29.3	180.5 ± 19.9	.432
ALT (U/L)^c^	33.6 (24.0‐97.0)	71 (22.6‐198.0)	.076
AST (U/L)^a^	92.2 ± 9.9	107.2 ± 11.1	.387
ALP (U/L)^a^, ^c^	191.7 ± 33.5	150.1 (64.5‐322.2)	.175
GGT (U/L)^a^	335.8 ± 87.7	274.3 ± 42.1	.473
TBIL (µmol/L)^a^	29.7 ± 4.4	19.4 ± 1.8	.017
CRE (µmol/L)^a^	67.6 ± 4.9	68.1 ± 4.3	.938
PT (s)^a^, ^c^	13.8 ± 0.5	11.9 (11.5‐14.4)	.024
AFP (ng/mL)^c^	2,575.4 (20.28‐30,722)	11,527 (1.86‐121,000)	.437
Child‐Pugh score^b^			.071
5	4 (50.0%)	12 (80.0%)	
6	0 (0.0%)	2 (13.3%)	
≥7	4 (50.0%)	1 (6.7%)	
Type of chemotherapy drug^b^			.177
1	2 (25.0%)	0 (0.0%)	
2	1 (12.5%)	3 (20.0%)	
3	5 (62.5%)	12 (80.0%)	
Usage of PVA/AGS^b^			1.000
No	8 (100.0%)	14 (93.3%)	
Yes	0 (0.0%)	1 (6.7%)	
Tumor response^b^	4 (100%)	13 (100%)	1.000
CR	0 (0%)	0 (0%)	
PR	1 (25.0%)	2 (15.4%)	
SD	1 (25.0%)	5 (38.5%)	
PD	2 (50.0%)	6 (46.2%)	
Postinterventional index^d^			
PLT (×10^9^/L)^a^, ^c^	108.6 ± 16.6	131.0 (31.5‐146.0)	.675
ALT (U/L)^c^	64.6 (29.4‐368.0)	178.0 (30.0‐1,030.1)	.039
AST (U/L)^a^, ^c^	167.4 ± 38.5	366.3 (31.0‐1,329.9)	.039
ALP (U/L)^a^, ^c^	170.1 ± 25.3	163.9 (98.0‐424.6)	.699
GGT (U/L)^a^	291.9 ± 73	306.4 ± 47.4	.864
TBIL (µmol/L)^a^, ^c^	40.3 ± 4.9	44.2 (22.3‐136.6)	.519
CRE (µmol/L)^a^, ^c^	68.8 (62.8‐93.3)	67.43 ± 4.47	.272
OS (mo)^e^	6.8 (0‐21.4)	5.1 (4.8‐5.3)	.055
PFS (mo)^e^	2.7 (0‐8.2)	2.3 (0.5‐4.1)	.064

Abbreviations: AFP, alpha‐fetoprotein; AGS, absorbable gelatin sponge; ALP, alkaline phosphatase; ALT, alanine aminotransferase; AST, aspartate aminotransferase; CRE, creatinine; GGT, glutamyl transpeptidase; HBsAg, hepatitis B surface antigen; ICG R15, retention rate of indocyanine green for 15 minutes; NA, not assessable; OS, overall survival; PD, progression disease; PFS, progress‐free survival; PLT, blood platelet; PR, partial response; PT, prothrombin time; PVA, polyvinyl alcohol; SD, stable disease; TBIL, total bilirubin.

a Mean ± SE.

b No. (%).

c Median (range).

d Clinical serum index after intervention within 1 week.

e Medium (95% CI).

### 3.4 Treatment strategy in patients with ICG R15 ≤ 10%

In patients with ICG R15 ≤ 10%, we propose a new indicator, the LDR, which is the ratio of the lipiodol dose (mL) to the maximum tumor diameter (cm), to describe the relationship between the lipiodol dose and the tumor load. Patients with ICG R15 ≤ 10% were categorized into a standard subgroup (n = 40), in which the patients received triple‐drug chemotherapy treatments with LDR values between 1 and 3, and a nonstandard subgroup (n = 16), in which the patients received other different treatment. The clinical characteristics and treatment analyses of these patients are shown in Table 4. We found that, when the baseline characteristics were comparable, patients who received TACE treatments with LDR values between 1‐3 and triple‐drug chemotherapy demonstrated better prognoses than those who received other TACE treatment options. The median OS (10.3 vs 5.8 months; 95% CI: 2.1‐18.5 vs 0.2‐11.4 months; *P* = .021) and PFS (3.3 vs 1.4 months; 95% CI: 0.1‐6.5 vs 0.7‐2.0 months; *P* = .046) were higher in the standard as compared to the nonstandard group (Figure 2E,F).

**Table 4:** Clinical characteristics and treatment analyses of ICG R15 ≤ 10% patients, grouped by LDR and chemotherapy drugs

Characteristic	1 ≤ LDR ≤ 3 with Triple‐drug chemotherapy (n = 40)	Others (n = 16)	*P* value
Age (y)^a^	45.7 ± 1.7	41.4 ± 2.9	.188
Gender^b^			.307
Female	2 (5.0%)	3 (18.8%)	
Male	38 (95.0%)	13 (81.3%)	
Maximal diameter of tumor (cm)^a^	9.9 ± 0.4	10.0 ± 0.6	.956
Number of tumor (s)^b^			1.000
Single	18 (45.0%)	7 (43.8%)	
Multiple	22 (55.0%)	9 (56.3%)	
Tumor distribution^b^			.771
Unilobar	20 (50.0%)	7 (43.8%)	
Bilobar	20 (50.0%)	9 (56.3%)	
Macrovascular invasion^b^			.676
Absent	5 (12.5%)	3 (18.8%)	
Present	35 (87.5%)	13 (81.3%)	
Extrahepatic metastasis^b^			.737
Absent	30 (75.0%)	13 (81.3%)	
Present	10 (25.0%)	3 (18.8%)	
Ascites^b^			.584
Absent or mild	38 (95.0%)	16 (100.0%)	
Moderate or severe	2 (5.0%)	0 (0.0%)	
HBsAg^a^			.426
Negative	7 (12.5%)	1 (4.3%)	
Positive	49 (87.5%)	22 (95.7%)	
PLT (×10^9^/L)^a^	208.0 ± 12.0	229.9 ± 19.7	.340
ALT (U/L)^a^, ^c^	44.5 (10.4‐169.2)	65.2 ± 10.8	.394
AST (U/L)^a^, ^c^	58.9 (19.2‐179.7)	77.5 ± 10.4	.599
ALP (U/L)^a^, ^c^	118.7 (62.0‐1682.5)	134.6 ± 12.1	.586
GGT (U/L)^a^, ^c^	177.0 (51.5‐560.8)	178.5 ± 23.2	.885
TBIL (µmol/L)^c^	16.1 (5.0‐50.1)	14.2 (8.2‐31.4)	.556
CRE (µmol/L)^a^, ^c^	74.2 ± 2.0	66.6 (42.4‐125.6)	.095
PT (s)^a^, ^c^	12.3 (10.8‐15.2)	12.8 ± 0.3	.586
AFP (ng/mL)^c^	5,055.0 (2.31‐121 000)	62,041.0 (2.26‐121 000)	.363
Child‐Pugh score^b^			1.000
5	33 (82.5%)	14 (87.5%)	
6	7 (17.5%)	1 (6.3%)	
≥7	0 (0.0%)	1 (6.3%)	
Dose of lipiodol (mL)^a^, ^c^	20.0 (8.0‐30.0)	13.6 ± 2.2	.047
Usage of PVA/AGS^b^			.421
No	35 (87.5%)	12 (75.0%)	
Yes	5 (12.5%)	4 (25.0%)	
Tumor response^b^	33 (100%)	15 (100%)	.705
CR	0 (0%)	0 (0%)	
PR	2 (6.1%)	1 (6.7%)	
SD	16 (48.5%)	5 (33.3%)	
PD	15 (45.5%)	9 (60.0%)	
Postinterventional index^d^			
PLT (×10^9^/L)^a^	139.0 ± 8.9	160.2 ± 17.1	.236
ALT (U/L)^a^, ^c^	115.0 (15.8‐984.9)	200.6 ± 36.2	.690
AST (U/L)^c^	246.2 (38.7‐1561.4)	176.15 (33.4‐1226.8)	.326
ALP (U/L)^a^, ^c^	137.0 (54.6‐2176.6)	174.2 ± 20.7	.711
GGT (U/L)^a^, ^c^	214.9 (82.7‐1004.5)	216.7 ± 24.4	.684
TBIL (µmol/L)^c^	30.1 (12.3‐74.9)	34.0 (14.0‐83.4)	.535
CRE (µmol/L)^c^	72.8 (42.9‐132.44)	65.9 (46.0‐109.4)	.060
OS (mo)^e^	10.3 (2.1‐18.5)	5.8 (0.2‐11.4)	.021
PFS (mo)^e^	3.3 (0.1‐6.5)	1.4 (0.7‐2.0)	.046

Abbreviations: AFP, alpha‐fetoprotein; AGS, absorbable gelatin sponge; ALP, alkaline phosphatase; ALT, alanine aminotransferase; AST, aspartate aminotransferase; CRE, creatinine; GGT, glutamyl transpeptidase; HBsAg, hepatitis B surface antigen; ICG R15, retention rate of indocyanine green for 15 minutes; NA, not assessable; OS, overall survival; PD, progression disease; PFS, progress‐free survival; PLT, blood platelet; PR, partial response; PT, prothrombin time; PVA, polyvinyl alcohol; SD, stable disease; TBIL, total bilirubin.

a Mean ± SE.

b No. (%).

c Median (range).

d Clinical serum index after intervention within 1 week.

e Medium (95% CI).

### 3.5 Validation of SBI

**Figure 3:**
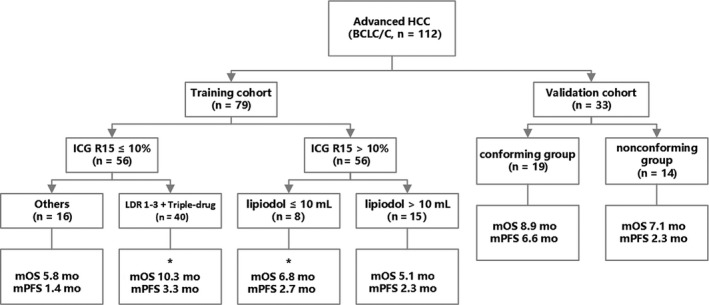
Grouping analysis and strategy diagram for transarterial chemoembolization treatment in Barcelona clinic liver cancer (BCLC) stage C hepatocellular carcinoma (HCC) patients. *Recommended treatment strategy. ICG, indocyanine green

**Figure 4:**
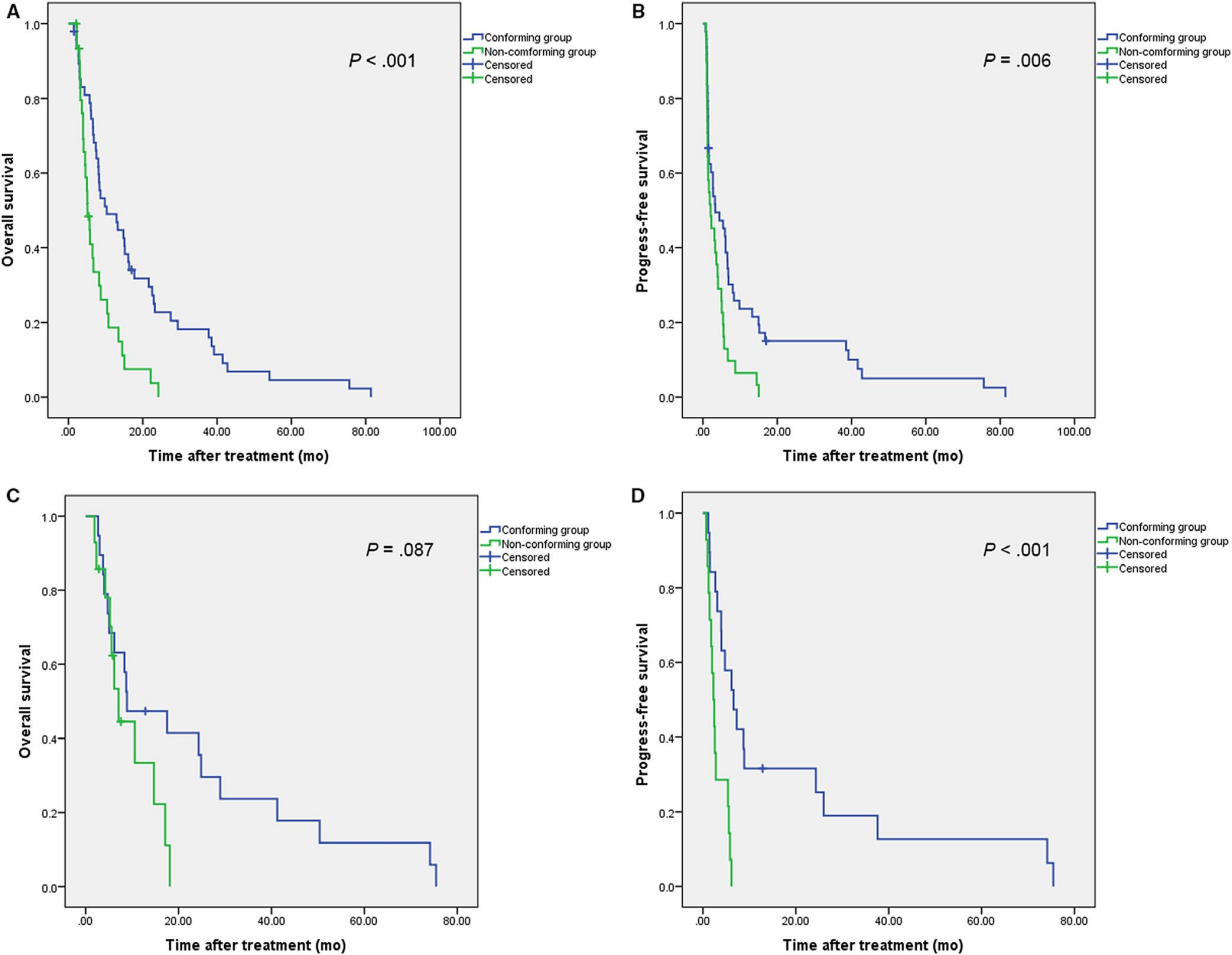
Kaplan‐Meier survival curves for patients conforming with the strategy based on indocyanine green (SBI) and those not conforming with the SBI. A, Overall survival (OS) in the training cohort; (B) progression‐free survival (PFS) in the in the training cohort; (C) OS in the validation cohort; (D) PFS in the validation cohort

Based on the above results on ICG R15 (strategy based on indocyanine green [SBI]) for BCLC stage C HCC patients, we propose the following TACE treatment strategy. Details of analysis were shown in Figure 3. For patients with ICG R15 > 10%, the lipiodol dose should be reduced to no more than 10 mL, and for those with ICG R15 values ≤ 10%, maintain the LDR value between 1 and 3 and use a triple chemotherapy drug regimen. For both the training cohort and the validation cohort, we then categorized the patients into a conforming group and a nonconforming group, based on whether their treatments conformed with the SBI. The survival analysis for the training cohort is shown in Figure 4A,B. Both the OS (median 10.3 months vs 5.1 months; 95% CI: 3.7‐16.9 months vs 3.8 −6.4 months) and PFS (median 3.3 months vs 2.1 months; 95% CI: 0‐6.9 months vs 0.2 −3.9 months) of patients in the conforming group were better than those in the nonconforming group (*P* < .001 and *P* = .006, respectively).

We then assessed the application of this strategy in the validation cohort. The survival analysis of the validation cohort demonstrated a similar prognosis tendency to that observed for the training cohort (Figure 4C,D). The median OS value was 8.9 months (CI: 0‐21.5 months) in the conforming group and 7.1 months (CI: 4.7‐9.5 months) in the nonconforming group (*P* = .087). The median PFS value was 6.6 months (CI: 0‐21.5 months) in the conforming group and 2.3 months (CI: 1.4‐3.1 months) in the nonconforming group (*P* < .001).

## 4 DISCUSSION

It is widely recognized that the BCLC staging roughly classify unresectable liver cancer patients into B and C stages.^14^ However, patients in BCLC stage C normally present with largely heterogeneous clinical characteristics, including liver function, tumor burden, vascular invasion, extrahepatic metastasis, and the presence of arteriovenous or arterioportal fistulas, which can lead to significantly different prognoses.^15^ The careful identification of advanced HCC patients who would benefit from the TACE treatments is very important to avoid overtreatment of nonrespondents.^16^ Thus, a number of researchers have developed models to predict the efficacy of TACE treatments.^17^, ^18^, ^19^, ^20^, ^21^ However, the existing models have generally been constructed for patients with BCLC stage B and are based on the pretreatment characteristics of the patients, without consideration to the recommended TACE treatment strategies. These models are not suitable for actual clinical application, as TACE is implemented through different techniques at different medical centers.^22^ The various treatment strategies consisting of the use of embolization agents, chemotherapy drugs, and the combination of therapeutic methods, affect therapeutic efficacy of the TACE treatments.^23^, ^24^, ^25^ Therefore, we designed this study with the aim of establishing a practical recommended strategy to better guide clinical practices and improve the therapeutic efficacy of TACE treatments advanced HCC patients.

Due to the high tumor burden, possible vascular invasion and unsatisfactory performance status of BCLC stage C liver cancer patients, the deterioration of liver function is one of the primary factors of poor prognoses.^26^, ^27^, ^28^ Moreover, given that TACE plays a role in the palliative settings of advanced liver cancer, the choice of treatment strategy should be based not only on the technical feasibility of the operation and the expected prognosis for oncotherapy but also on the possible survival benefits, which may outweigh the survival disadvantages due to worsening liver function after treatment.^29^ Therefore, we considered using ICG R15 as an indicator of liver function to separate these patients. The ICG R15 cutoff value of 10% was first proposed by Makuuchi et al and has been commonly used in clinical practice as a reference value for right hepatectomy and larger surgeries in patients with bilirubin ≤ 17.1 µmol/L.^7^ In our study, we found that an ICG R15 value of 10% could also be used as a threshold for evaluating the liver function of patients receiving TACE treatments and was consistent with the Child‐Pugh score, ascites, levels of AST, ALB, TBIL, and other indicators of liver function. Compared with patients having ICG R15 > 10%, those with ICG R15 ≤ 10% had no significant differences in tumor burden characteristics, including maximum diameter of tumors, number of tumors, the involvement of vascular invasion, and extrahepatic metastasis, tumor remission rate, or PFS values, although patients with ICG R15 ≤ 10% did had better OS as compared to those with ICG R15 > 10% (median 8.7 months vs 5.1 months; 95% CI 3.1‐14.3 months vs 3.4‐6.8 months; *P* = .005). These results suggest that among advanced liver cancer patients with ICG R15 > 10%, the reduction in survival might be caused by liver function deterioration rather than tumor progression. Therefore, for this patients with poor liver function, our primary treatment plans should emphasize protecting liver function over efficiently killing the tumor.

Our study showed that, among patients with ICG R15 > 10%, those patients treated with >10 mL lipiodol tended to have poorer survival outcomes than those treated with ≤10 mL lipiodol but these differences were not statistically significant. Although patients treated with higher doses of lipiodol tended to have larger tumors, the levels of ALT and AST in these patients were significantly higher after TACE treatment than those of patients treated with lower doses of lipiodol; however, there were no significant differences in the ALT and AST levels between the two groups before TACE treatment. In contrast, patients treated with lower lipiodol doses had higher levels of TBIL before TACE treatment, but the levels of TBIL were similar between the two groups after TACE treatment. These results suggest that patients treated with higher doses of lipiodol had more severe liver function deterioration after TACE treatment. In addition, it is worth noting that, even at the cost of worsening liver function, patients treated with higher doses of lipiodol did not experience better tumor remission rates. Shalimar et al indicated that patients with higher ICG retention and ICG plasma disappearance rates had higher risks of liver failure after TACE treatment.^9^ For these high‐risk patients, an appropriate reduction in lipiodol doses during surgery could protect their liver function. Reducing the lipiodol dose not only prevented reductions in the tumor remission rates and therapeutic efficacy but also promoted posttreatment recovery by providing the possibility of receiving other treatments, thereby prolonging survival time and improving quality of life of the patients.

Unlike for patients with ICG R15 > 10%, for whom there were a few treatment options are available due to their poor liver function, for patients with ICG R15 ≤ 10%, higher doses of lipiodol could be used to more efficiently attack the cancer cells as their liver function are generally better. However, the use of unrestricted levels of medication should still be opposed.

Regarding the strong heterogeneity observed in HCC, a simple cutoff value for the lipiodol dose would be overgeneralized; therefore, we formulated a new simple indicator, the LDR, in an attempt to identify the optimal lipiodol dose for TACE treatments. A study performed by Shi Ming et al showed that chemotherapeutic drugs also play irreplaceable roles in TACE treatment.^23^ In actual clinical practice, the application of chemotherapy drugs varies greatly. Our study suggested that, when the LDR value was maintained between 1 and 3 and a triple chemotherapeutic drug regimen combining anthracyclines (doxorubicin or epirubicin), platinum (cisplatin, lobaplatin, or carboplatin), and fluorouracil (floxuridine or fluorouracil) was prescribed, both the OS (median 10.3 months vs 5.8 months; 95% CI 2.1‐18.5 months vs 0.2‐11.4 months; *P* = .021) and PFS (median 3.3 months vs 1.4 months; 95% CI 0.1‐6.5 months vs 0.7‐2.0 months; *P* = .046) were superior than when other therapeutic options were given. Therefore, this treatment strategy was included in our recommended SBI for patients with ICG R15 ≤ 10%.

The above findings were validated using a cohort of 33 patients, for which the patient characteristics were similar to those of the training cohort. Our analyses confirmed the reliability of our treatment strategy proposed. Interestingly, whether patients had good or poor liver function, or whether or not their treatments conformed with the SBI, no differences in tumor remission rates were observed. This result suggests that, for advanced liver cancer, the tumor remission rate may not be directly related to patient survival, which is commonly observed in targeted therapies for advanced liver cancer.^30^ During TACE treatment, some patients may have survival benefits, even under conditions where their tumor responses are not satisfactory.

There are several limitations associated with this study worth mentioning. First, as a retrospective analysis, selection bias was inevitable. To reflect the tumor and liver function status of patients as comprehensively as possible, this study selected patients with complete imaging data and ICG examinations before the initiation of the TACE treatment and possibly excluded patients with very poor economic conditions or emergencies caused by tumor rupture. Second, only a small number of eligible patients were included. The 112 patients were divided into a training cohort and a validation cohort, and the training cohort (79 cases) was further divided into four groups, according to ICG R15 and the intraoperative treatment strategy, resulting in only 8 cases in the smallest group, which may have to a certain extent lead to deviations and bias in patients selection. Third, we used the largest diameter of the tumors to determine the LDR rather than the total diameter or up to two of the target lesions, as in the mRECIST criteria,^12^ which could underestimate the tumor burden of patients with multiple tiny lesions. Further exploration and future research are necessary to identify a more accurate indicator for the simple estimation of tumor burden. Fourth, ICG examination is still not commonly used in many medical centers, which restrict the universality of this SBI.

In conclusion, the selective application of TACE treatments can convey survival benefits to BCLC stage C HCC patients. However, due to the high tumor burden, extensive tumor involvement, poor liver function, and performance status of advanced HCC patients, a practical and reliable TACE treatment strategy guideline is necessary to standardize treatment for better efficacy. The SBI we are proposing is clinically operable and can effectively promote curative effects, better protect liver function, improve quality of life, and prolong the survival of BCLC stage C HCC patients. However, the SBI still requires further improvements and verification with larger sample sizes in future studies.

## CONFLICTS OF INTEREST

None declared.

## INFORMED CONSENT STATEMENT

Informed consent was not required for this study as the analyses were from anonymous clinical data that were obtained after each patient agreed verbally to undergo treatment. Individuals cannot be identified based on the data presented.

## Data Availability

The study data are available from the Sun Yat‐Sun University Cancer Center Institutional Data Access/Ethics Committee for researchers who meet the criteria for access to confidential data.
